# User-Operated Audiometry Project (UAud) – Introducing an Automated User-Operated System for Audiometric Testing Into Everyday Clinic Practice

**DOI:** 10.3389/fdgth.2021.724748

**Published:** 2021-10-07

**Authors:** Christos Sidiras, Raul Sanchez-Lopez, Ellen Raben Pedersen, Chris Bang Sørensen, Jacob Nielsen, Jesper Hvass Schmidt

**Affiliations:** ^1^Faculty of Engineering, The Maersk Mc-Kinney Møller Institute, University of Southern Denmark, Odense, Denmark; ^2^Interacoustics Research Unit, Kongens Lyngby, Denmark; ^3^Hearing Systems Section, Department of Health Technology, Technical University of Denmark, Kongens Lyngby, Denmark; ^4^Department of Clinical Research, Faculty of Health Science, University of Southern Denmark, Odense, Denmark; ^5^OPEN, Open Patient Data Explorative Network, Odense University Hospital, Odense, Denmark; ^6^Research Unit for ORL-Head and Neck Surgery and Audiology, Odense University Hospital and University of Southern Denmark, Odense, Denmark

**Keywords:** audiometry, hearing loss, user-operated, automated, hearing aid, hearing test, telehealth

## Abstract

Hearing loss is the third leading cause of years lived with disability. It is estimated that 430 million people worldwide are affected, and the number of cases is expected to increase in the future. There is therefore increased pressure on hearing health systems around the world to improve efficiency and reduce costs to ensure increased access to quality hearing health care. Here, we describe the User-Operated Audiometry project, the goal of which is to introduce an automated system for user-operated audiometric testing into everyday clinic practice as a means to relieve part of this pressure. The alternative to the existing referral route is presented in which examination is executed via the user-operated system. This route is conceptualized as an interaction between the patient, the system, and the hearing care professional (HCP). Technological requirements of the system and challenges that are related to the interaction between patients, the user-operated system, and the HCPs within the specific medical setting are discussed. Lastly, a strategy for the development and implementation of user-operated audiometry is presented, which includes initial investigations, a validation study, and implementation in a real-life clinical situation.

## Introduction

Hearing loss is the third leading cause of years lived with disability (1). The World Health Organization estimates that 430 million people worldwide live with a disabling hearing loss and one-third of older adults (>65 years) are affected by this condition (2). The annual cost for untreated hearing loss is estimated by the WHO to be 980 billion USD globally. Recent studies have also highlighted hearing loss as being one of the greatest modifiable risk factors for cognitive decline, dementia, and depression later in life (3–6). Since the most common type of hearing loss is associated with age and given that the percentage of people above the age of 65 is increasing (2), it is expected that the number of people with hearing loss will also increase (1). There is therefore increased pressure on hearing health systems around the world to improve efficiency and reduce costs to ensure increased access to quality hearing health care.

Treatment of hearing loss often involves the examination of hearing function, the selection and fitting of hearing aids, and the evaluation of hearing-aid performance. As part of the initial assessment, the pure-tone audiometry is time-consuming, as testing time may exceed 20 min (7, 8) and seems to be the bottleneck for further clinical decisions in the current health care system. According to data from the Danish Health and Medicine Authority (9), the current waiting time for the examination in the public system is up to 86 weeks. Currently, this assessment requires the presence of a hearing care professional (HCP). Although pure-tone audiometry is not the only examination of the initial assessment, it is probably the most time-consuming part. The development of a user-operated audiometry system, that is, one that does not require the presence of a HCP, will arguably help to free resources needed in the initial assessment procedure.

Here, we describe the User-Operated Audiometry (UAud) project, the goal of which is to introduce an automated system for user-operated audiometric testing into everyday clinical practice. Importantly, the user-operated system is not intended to replace the audiological assessment done by an HCP, but rather to offer an alternative to manual audiometric testing when applicable. Furthermore, the UAud project focuses on the user-operated diagnostic examination, in the clinical environment and with calibrated clinical devices. Although these approaches, such as asynchronous tele-audiology (10), have shown their potential as screening tools and likely impact for reducing the global burden of hearing loss, the UAud scope is on the more efficient use of human resources in the hearing healthcare services (11). It is therefore expected that the user-operated hearing assessments will reduce the clinical hours spent on air conducted pure-tone tests by a significant amount, freeing up time that can be spent better by the HCP on counseling the patient on using hearing aids and/or to see more patients throughout the day.

The purpose of this perspective article is (a) to briefly review previous relevant related work about user-operated audiometry, (b) to describe the scope and focus of the UAud project, (c) to create a perspective on the challenges and possible barriers related to the inclusion of the new examination paradigm, and (d) to present a strategy for addressing these challenges and effectively implement user-operated audiometry in the daily practice.

### Previous Research

The opportunities of automated audiometry have been explored since the beginning of the computer age (12) and has been widely used for research purposes (13). Further, automated audiometry is implemented for medical purposes, though limited to screening (14, 15). Recently, research efforts have shown promising results toward its potential diagnostic use (16–18), both in the clinic and as an opportunity for implementing tele-audiology (19, 20). In a systematic review, Mahomed et al. (21) suggested that automated pure-tone audiometry provides an accurate measure, but validation is still needed for specific cases such as difficult-to-test populations. In a more recent review, Shojaeemend and Ayatollahi (22) concluded that automated audiometry produces clinically acceptable results compared with traditional audiometry.

Considerable research contributions have been focused on the Automated Method for Testing Auditory Sensitivity (AMTAS®) test, a single-interval, forced choice method (yes-no paradigm) task with an adaptive algorithm for pure-tone detection thresholds (23). This test is intended to be used in-the-clinic with standardized diagnostic equipment but in an asynchronous user-operated approach. The HCP instructs the patient and supervises the accuracy of the results, but they do not need to be present while the test is being carried out. A series of studies has explored its accuracy and validity in clinical settings in children, adults, and elderly populations (23–28). Overall, the use of user-operated audiometry using medical equipment and a controlled environment shows promise for the implementation of AMTAS® in the current clinical practice.

### Automated Audiometric Tests Beyond Pure-Tone Audiometry

The automatization of presenting pure tones to a patient while concurrently analyzing the patient's responses does not present many technical challenges and has the potential to be included in a user-operated version suitable for a broad part of the population. However, the implementation of other user-operated audiological tests such as speech audiometry is more challenging. Research efforts have been made in the direction of automatizing speech-in-noise recognition (29, 30), and the development of self-scoring multilingual speech tests (31, 32). These tests still need a careful selection and validation of the speech material, and their ecological validity is limited. Either the so-called sentence-matrix tests [first introduced by Hagerman (33)] or the digit-triplet test [first introduced by Smits et al. (34) as a screening speech intelligibility test by telephone] are both affected by the same drawbacks. On one hand, the development and validation of the speech material in different languages is easier than for other tests (e.g., hearing in noise test) but it still requires substantial efforts. On the other hand, are the speech reception thresholds obtained using these tests are unrealistic since the speech stimulus is cognitively undemanding (fixed structured sentences or digits) and it is presented as a closed set (the patient has only a limited number of possible responses). Therefore, this argues against the introduction of a standardized test for assessing speech recognition abilities in noise for worldwide implementation.

The assessment of the patient's ability to extract the essential features of the speech signal may lend itself to a more practical solution. The Audible Contrast Threshold (ACT™) is a new clinical test measuring spectro-temporal modulation sensitivity, in which the subject's task is to discriminate between spectro-temporally modulated noise and non-modulated noise. The idea that the similarity of the spectro-temporal characteristics of the modulated stimuli and real speech would make sensitivity to spectro-temporal modulations a good predictor of speech-in-noise performance has been investigated by several research groups (35–37) with promising results. Subsequent research (38) indicated that the spectro-temporal modulation test used was too difficult for about 1/3 of the large clinical population tested. This problem was eventually solved by Zaar et al. (39), who further went on to show how spectro-temporal modulation detection thresholds predict aided speech-in-noise recognition in an ecologically valid scenario, as well as the benefit from using noise reduction in hearing aids (40). This research (39, 40) forms the basis of the clinical ACT™ test, which thus is a suprathreshold audiometric proxy for speech-in-noise testing with hearing aids measured with a language-independent stimulus. A further advantage of the ACT™ test is that it lends itself well to automatic user-operated implementation.

## Incorporating User-Operated Audiometry Within the Existing Clinical Procedure

**Figure 1:**
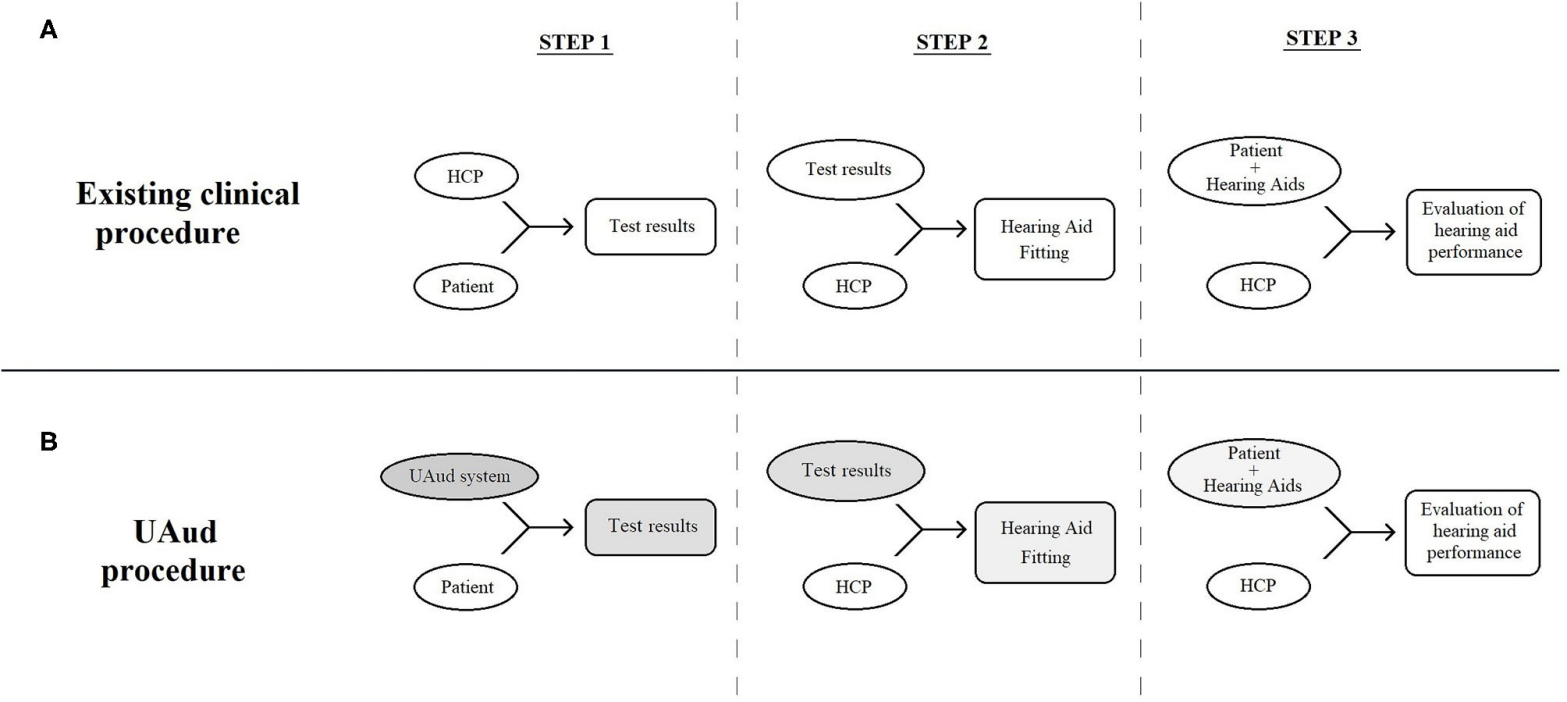
Schematic representation of the 3-step existing **(A)** and user-operated audiometry **(B)** procedure, consisting of three parties, i.e., the patient, the user-operated system and the HCP. The user-operated examination affects step 1, and consequently steps 2 and 3, as medical decisions are based on the results of the former.

The existing clinical procedure for hearing-aid fitting consists of three main steps. Step 1: examination of hearing–audiometry, step 2: initial fitting of the hearing aids, and step 3: evaluation of hearing aid performance and re-adjustment if needed (see Figure 1A). Normally, all three steps are carried out by an HCP. The whole procedure requires the involvement of two parties (patient and HCP) who interact throughout all three steps.

The UAud project explores an alternative procedure by freeing the HCP from the major part of the first step (i.e., the audiometric tests, excluding the otoscopy and anamnesis) and instead introducing the user-operated system, while all the tasks and responsibilities of the HCP in steps 2 and 3 remain unchanged. This new procedure can be conceptualized as a human-digital system-human interaction (i.e., the patient, the user-operated system and the HCP, respectively). The three parties interact more than once and in more than one way (see Figure 1B), but the interaction is more complicated compared to the existing clinical procedure. The user-operated examination affects step 1, and consequently steps 2 and 3, as the practical hearing-aid fitting is based on the results of the former. Further and beyond these effects, there are less obvious ones. The HCP's reservations and skepticism may negatively affect both hearing-aid fitting and evaluation/re-adjustment (13). This may happen if either the patient or the HCP is skeptical of the accuracy of the hearing examination.

In order to better understand the dynamics of the 3-parties system (patient, user-operated system, and HCP), it seems useful to have a detailed perspective of the relevant characteristics of the parts, (i.e., humans and digital technology). Humans are goal-oriented and goal-directed (41). They learn quickly, have powerful selective attention, can be comparatively easily excited and get focused on something they find interesting, either feature or process (42). On the other side, humans are easily distracted, lose their interest quickly, or even give up if they get disappointed, confused, or tired. They have limited cognitive resources [e.g., working memory and attention; (43)], and they are often cognitively or emotionally biased (44).

Digital technology comes with its own pros and cons. Compared to humans, it is typically governed by clearer and known rules, so it can be standardized comparatively easily, and still offers some versatility through settings in the system. On the other hand, digital technology tends to become less adaptable after its release, and paradigm changes may require completely new technology to be developed, whereas a human might only need short retraining. Thus, every detail on every process and all potential pitfalls/bugs, must be thought of and addressed in advance before implementation. Excluding an arguably significant, still not relevant to the present study, part of current technology, i.e., artificial intelligence, one could argue that humans learn while technology is set.

Bringing together humans and digital technology is a challenge on its own, as large differences between them may negatively affect their interaction. Making digital technology more user-friendly and measuring the quality of the interaction is a wide field of study of its own and has a fast-growing body of research output in the last few decades. Indicatively, a search in Scopus with keywords [“usability” AND “technology”] yields 717 review studies alone (searched on 8-March-2021), while there is an increasing interest on usability for health evaluation and intervention tools [for reviews see (45–47)]. In the UAud project, the quality of the human-system interaction is crucially important, as deviations from optimum may affect not only the examination, i.e., the first step, but also the whole procedure.

Insights on the effects of the introduction of user-operated audiometry can be gained by comparing with audiometry operated by an HCP. Focus should be given on those aspects of human and digital technology that are crucial for the quality of the hearing examination results. In Table 1 the main advantages of the HCP-operated test are in line with the disadvantages of the user-operated audiometry and vice versa. While the user-operated system can make use of the computation intelligence for analyzing the quality of the test results and apply a well-defined protocol to obtain the patient's hearing thresholds, the HCP-operated examination has the advantage that the professional can supervise and adapt the procedure to patient if needed (23), which is particularly important in certain populations (e.g., children and people with mild to moderate cognitive impairments).

**Table 1:** Primary examples of the advantages and drawbacks of manually- and user-operated audiometry procedures identified in the UAud project.

	**HCP operated audiometry**	**User-operated audiometry**
Advantages	- The HCP can start the test when the listener has understood the task - Adapt the procedure and instructions to the individual - Constant supervision and observation - HCPs can trust their own actions	- Indirect measures gathered during the test: Reaction time - The procedure is well-defined, and all listeners are tested identically - Objective metric of the quality of the measurement
Drawbacks	- The protocol and criteria can vary from one HCP to other - The experience of the HCP may affect the measurement	- The user has to learn the task alone - The procedure cannot (a priori) be adapted to the individual - The HCP requires evidence to trust that patients have consistent response criteria

*The table is the result of discussions among some of the researchers involved in the initial investigations of the assumption that the user-operated test will be performed by the patient alone*.

## Scope of the Project

The UAud project aims to explore the possibilities of implementing user-operated audiometry in everyday clinical practice. The technological progress on automated audiometry of the past decades will be further developed, evaluated, and implemented in the clinic in the form of a system for user-operated testing of air-conduction pure-tone audiogram and ACT™. As the motivation behind the project is to free work hours from HCPs dedicated to the hearing examination, the system must be handled by the patient ideally without any supervision. All actions taken by the HCP (11, 23) such as preparation for and carrying out the examination and monitoring the behavior of the patient, must be executed in the absence of the HCP.

The requirements for successful implementation of the user-operated audiometry system are:

- The establishment of the necessary software, hardware, and testing environment.
- Acceptable usability and user experience of the system's software and hardware.
- Strategies to deal with situations such as patients needing further instructions or other considerations (patient's presenting tinnitus).
- The system must be designed to ensure that the patient's attention is adequately focused on the task during the examination.
- Optimal time efficiency, that is, the test must be accurate and reliable while avoiding causing patient fatigue due to extensive test time.
- Effective supervision, that is, the system must assess the quality of the examination, and give recommendation for further testing if necessary.

Further, the UAud project must account for the specific setting in which the system will be implemented. The setting includes (a) the medical context, that is, hearing health care and specifically the audiological examination (step 1 in Figure 1), (b) the specific use of the test results, that is, informing hearing intervention (as depicted in steps 2 and 3), (c) the people involved, that is, audiological patients, many of which are elderly, and HCPs, and (d) the fact that the patient will be examined alone, i.e., without the supervision of a HCP. Factors that must be accounted for include:

- Emotional factors of the patient at different stages of the process: (a) before the examination (e.g., are his/her feelings positive or negative toward the use of user-operated tests?, is he/she confident that the examination will go well?), (b) during the examination (e.g., does he/she feel confident with this approach or does he/she experience frustrations?), and (c) after the examination (e.g., does he/she feel that the examination went well and the results are accurate?). These factors may affect the examination itself, and further influence the HCP's confidence in the validity of the examination and consequently his/her decisions in the rehabilitation process.
- The lack of the positive effect on the patient due to presence of a human medical expert during the examination. This may partially affect the trust, knowledge, regard, and loyalty that characterizes the patient-HCP relationship (48).
- Intrinsic factors of the patient: computer skills, biases against technology, cognitive decline, and comorbidities.
- HCPs, as all clinicians, are very cautious and demand high quality and evidence before accepting a new examination paradigm as part of their everyday clinical practice (13). It is reasonable to assume that HCPs' skepticism will be pronounced in the case of a user-operated examination.
- HCPs work as a community, at least to a degree (13). Experiences shared among colleagues concerning clinical practices and interventions affect how new practices are accepted (13), and this may play a role in how user-operated audiometry will be received.
- The potential disruption to standard operating procedures, e.g., changing appointment times, effects on allocation of human and physical resources.

## Toward the User-Operated Audiometry Implementation

The success of the UAud project relies on the extent to which the user-operated audiometry system meets the aforementioned requirements and is accepted by both patients and HCPs, enabling wide adoption in audiological practice. Here, a research plan is presented which includes (1) initial investigations, (2) a validation study where this system will be evaluated in a randomized clinical trial, and (3) implementation in a semi-large scale within real-life clinical conditions.

### Initial Investigations

The initial investigations consist of (a) prototyping and user-interaction design of the user-operated solutions, (b) evaluation of the equipment and testing environment toward a usable system, (c) implementation and validation of a user-operated ACT™ test, and (d) identifying barriers on implementation. At this early stage, a detailed record of patients' and HCPs' concerns that will inform the whole project's research plan is needed. This will include semi-structured interviews (49) and questionnaires delivered to patients and HCPs. Further, specific research questions will be addressed by running “proof of concept” studies that will help preparations of the validation and implementation and address concerns raised by patients and HCPs. Some research questions will be part of these studies:

- How can the design of the user interface maximize the internal/external motivation and minimize distractions?
- How do the patients interact with the system when doing a new audiometric test?
- Which is the preferred paradigm for assessing the ACT™ in a user-operated test?
- What is the effect of the user (instead of expert) placing of headphones on audiometry and ACT™? How to ensure its quality?
- What is the most effective design for the set of instructions required for handling both software and hardware? How can the system be designed in terms of both content and mode (e.g., text, audio, video, images, pictograms) to guarantee that a majority of patients can successfully complete the test?

### Validation Study

Hearing rehabilitation entails different types of interventions. Part of these interventions include the hearing-aid selection, provision, and fitting, which make use of the individual audiometric thresholds. The purpose of the validation study is to demonstrate that the quality of the intervention is not influenced by the modality of the hearing test (i.e., user-operated vs. manual audiometry). A randomized double blind clinical trial will be conducted on two groups of adult patients with a treatable hearing loss (125 in each group). HCPs will provide the intervention based on audiometric thresholds without knowledge of the modality used to obtain them. Two months after the hearing aid fitting, patients will be examined with a test battery which will include self-report outcome measures and an examination of speech intelligibility.

### Implementation

The UAud system will be implemented in a real-life situation, involving the allocation of resources for a new workflow in the selected clinics. The implementation of user-operated audiometry will be carried out at Odense University Hospital in Odense and Svendborg, as well as at two private ENT clinics. It will include a total of at least 250 patients. This is probably the most challenging part of the project, as there will be a need for clinicians adopt the user-operated audiometry as part of their daily work. Therefore, an effective change in the hearing healthcare service should involve different phases (50). First, the hearing care professionals have to be aware of the innovation. Second, there should be an understanding that the user-operated audiometry is indeed an opportunity to improve the person-centered service. Third, the barriers explored in the initial investigation have to be addressed so the HCPs can accept the change with a positive attitude. And forth, the actual implementation of the user-operated audiometry in the clinical practice, the confirmation of its benefits and the integration of the service in the clinical setup. An implementation manual will be published describing the user-operated audiometry as well as the limitations and criteria for its correct and efficient use.

## Author Contributions

All authors have made contributions to the manuscript, participated in the conceptualization, reviewed, and agreed with the final version of the manuscript.

## Funding

This work was supported by Innovation Fund Denmark Grand Solutions 9090-00089B and the William Demant Foundation.

## Conflict of Interest

RS-L is employed by Interacoustics A/S. The remaining authors declare that the research was conducted in the absence of any commercial or financial relationships that could be construed as a potential conflict of interest.

## Publisher's Note

All claims expressed in this article are solely those of the authors and do not necessarily represent those of their affiliated organizations, or those of the publisher, the editors and the reviewers. Any product that may be evaluated in this article, or claim that may be made by its manufacturer, is not guaranteed or endorsed by the publisher.
